# Mistrust of the medical profession and higher disgust sensitivity predict parental vaccine hesitancy

**DOI:** 10.1371/journal.pone.0237755

**Published:** 2020-09-02

**Authors:** Rebekah Reuben, Devon Aitken, Jonathan L. Freedman, Gillian Einstein

**Affiliations:** 1 Department of Psychology, University of Toronto, Toronto, Ontario, Canada; 2 Dalla Lana School of Public Health, University of Toronto, Toronto, Ontario, Canada; 3 Rotman Research Institute, Toronto, Ontario, Canada; 4 Tema Genus, Linköping University, Linköping, Östergötland, Sweden; Emory University, School of Public Health, UNITED STATES

## Abstract

Despite overwhelming evidence that vaccines are safe and effective, there has been a rise in vaccine hesitancy and refusal leading to increases in the incidence of communicable diseases. Importantly, providing scientific information about the benefits of vaccines has not been effective in counteracting anti-vaccination beliefs. Considering this, better identification of those likely to be vaccine hesitant and the underlying attitudes that predict these beliefs are needed to develop more effective strategies to combat anti-vaccination movements. Focusing on parents as the key decision makers in their children’s vaccination, the aim of this study is to better understand the demographic and attitudinal predictors of parental vaccine hesitancy. We recruited 484 parents using Amazon MTurk and queried their attitudes on childhood vaccination, level of education, age, religiosity, political affiliation, trust in medicine, and disgust sensitivity. We found three main demographic predictors for parental vaccine hesitancy: younger age, lower levels of education, and greater religiosity. We also found vaccine hesitant parents to have significantly less trust in physicians and greater disgust sensitivity. These results provide a clearer picture of vaccine hesitant parents and suggest future directions for more targeted research and public health messaging.

## Introduction

Vaccination has greatly reduced the burden of infectious diseases worldwide, and is an essential tool for public health [1]. Yet despite clear scientific evidence that vaccines are safe and effective, vaccine hesitancy and refusal has increased [2–3]. As a result, there has been a greater incidence of communicable diseases, such as measles and pertussis, in recent years [4]. The situation is so serious that the World Health Organization identified vaccine hesitancy as one of the ten greatest threats to global health (WHO, 2019). In light of the recent COVID-19 pandemic, the need for confidence in vaccines is even more crucial [5].

Attitudes towards vaccination range from active acceptance to complete refusal of all vaccines, while vaccine hesitant individuals form a heterogeneous group along this spectrum [6]. Prior research has aimed to identify demographic correlates of vaccine hesitancy, however a consistent profile across studies has yet to emerge. Religious beliefs are commonly cited as a reason for non-compliance with vaccination in the U.S. [7], and several studies have focused on vaccine refusal amongst specific religious groups. The effect of religiosity in general, irrespective of specific religious groups, has been less examined, although a mixed-sex sample of U.S. parents has found that greater religiosity was associated with higher rates of vaccine refusal [8]. Political ideology has also been proposed as a potential predictor of vaccine hesitancy. However, results from U.S. samples have been mixed, with some linking conservatism to greater vaccine hesitancy [9], while others determining an association with the extreme poles of both conservative and liberal ideologies [10]. Findings on education level have also been mixed, with a national sample of Canadian parents supporting an association between lower levels of education and vaccine concerns [11], while a review of U.S. medical records found vaccine refusers were more likely to reside in well-educated areas [12]. Although these findings provide some insight, they range across a variety of definitions of vaccine hesitancy, cohorts, and vaccine-related outcomes, which may provide further rationale for these inconsistencies. Thus, continued research on demographic predictors is needed to determine a clearer picture of individuals more likely to be vaccine hesitant.

Vaccine hesitancy is often explained as being due to a lack of knowledge or a miscalculation of risk [13]. However, the use of corrective information to combat vaccine hesitancy has been ineffective [14]. Providing corrective information has even produced a ‘backfire effect’, with a mixed-sex sample of U.S. parents indicating a reduced intent to vaccinate after receiving corrective information [15–16]. Therefore, simply providing factual information about the risks and benefits of vaccines does not seem to be an effective strategy to reduce vaccine hesitancy. An inquiry of the underlying attitudes behind vaccine hesitancy is needed to develop effective public health campaigns and reduce vaccine refusal [17].

The finding that providing evidence-based information is ineffective suggests that those who are vaccine hesitant do not accept the information. Risk-focused interventions rely on the assumption that corrective information will be trusted, and the lack of efficacy may point to a focus on mistrust, rather than perceived risk, as a major motivator of vaccine hesitancy [13]. In a recent review, mistrust of doctors, government sources, and pharmaceutical companies were commonly reported reasons for vaccine hesitancy [18]. Moreover, mistrust in science and the medical profession is known to play an especially important role in parental vaccination decisions [19–20]. Parents with lower rates of vaccinating their children reported less trust in physicians, nurses, pharmacists, and health clinics as sources of information [21]. Particularly, trust in pediatricians was a pivotal theme in a qualitative assessment of new mothers’ decisions to vaccinate their children [22].

Lack of trust in medicine and physicians has important corollary actions that shape parental attitudes towards vaccination. Parents who lack trust in their child’s doctor are more likely to consult the internet to obtain vaccine information, and parents who consult the internet for vaccine information are more likely to be vaccine hesitant and refuse vaccination for their child [23]. Posts about the dangers of vaccines are prevalent and easily accessible online. It has been reported that 43% of hits for ‘vaccination’ and ‘immunization’ on top search engines contain anti-vaccination material [24]. Many of these sites may reinforce mistrust in doctors, as commonly found themes include allegations that doctors are misinformed about vaccines, that doctors themselves do not trust the vaccines they administer, or that doctors are protected from liability for harm caused by vaccines [25–28]. Furthermore, anti-vaccination sites focus on emotional appeals, including personal testimonials using pictures and stories of children allegedly harmed by vaccines, threatening needle imagery, and calls for responsible and ethical parenting through standing against harmful vaccinations [24–25, 28].

Some research has utilized the emotional approach taken by anti-vaccination sites to alter vaccine hesitant attitudes. In one study, 265 university students between the ages of 19–36 (86.8% women) were provided with a description of a fictitious vaccine along with fictitious narratives from those vaccinated, and manipulated the frequency of vaccine-related adverse events reported in these narratives. They were also provided with the credibility of the website (anti-vaccination agenda vs neutral health forum), and the credibility of the statistical information on the occurrence of adverse events. The results showed that the narratives with higher frequencies of vaccine adverse events led to a greater perception of risk. However, there was no effect of manipulations of credibility on risk perception, either for the website hosting the narratives or the statistical information [29]. Taken together, this suggests that narrative accounts of vaccine-related adverse events create a strong bias towards perceiving risk, irrespective of the credibility of the website hosting the narratives or the reliability of statistical information provided.

Emotional appeals emphasizing the risks of not vaccinating have also been used to reduce vaccine hesitancy [14]. In this study, 315 participants from Amazon Mechanical Turk (MTurk) (50.8% women; mean age = 35.44 years, SD = 11.60) were randomly assigned to two separate conditions. In a condition called ‘disease risk’, participants were presented with a narrative from a mother whose child contracted the measles, photographs of children with measles and rubella, and warnings on the importance of vaccination. In a condition called ‘autism-correction’, participants were given information summarizing research showing that vaccines do not increase the risk of autism. Participants in the disease risk condition showed a significant reduction in vaccine hesitancy compared to corrective and control conditions. This effect was found even among those who reported higher levels of vaccine hesitancy at baseline, suggesting that the use of emotional appeals often seen on anti-vaccination sites may be successfully employed to, instead, counteract vaccine hesitancy.

Another common theme on anti-vaccination sites is the notion that vaccines are artificial and contain toxic, poisonous, or dangerous ingredients, as opposed to ‘natural’ products or homeopathic medicine [25, 27–28, 30–31]. Considering this, it has been suggested that vaccine hesitancy is rooted in a ‘health purity attitude’ that functions as protection from contamination and causes an affective disgust response [32]. Differences in disgust sensitivity, or the frequency and intensity at which individuals experience disgust, have been examined in relation to a variety of political and social attitudes. Higher levels of disgust sensitivity have been associated with disapproval of gay people [33], in-group preference and ethnocentrism [34], and social conservatism [35]. Furthermore, recent studies in undergraduates and the general population have linked higher levels of disgust sensitivity with negative attitudes towards vaccination [32, 36], suggesting this may be a motivating factor for vaccine hesitancy.

Numerous studies have explored vaccine hesitancy in relation to a variety of demographic and attitudinal predictors and across a range of groups and approaches. We were interested in exploring vaccine hesitancy in parents specifically–as they are a critically important group in making vaccine-related decisions [37]. This study investigated the association between vaccine hesitancy and demographics factors, trust in medicine, and disgust sensitivity. We hypothesized that vaccine hesitancy would be strongest in parents with less trust in medicine and higher pathogen disgust sensitivity.

## Methods

### Participants

Our dataset included 522 participants who were recruited via Amazon MTurk. Participants were excluded if they were not (1) parents, (2) citizens of the United States, Canada, or the United Kingdom, or (3) did not answer the parental vaccine hesitancy scale, trust in medical profession items, and the disgust sensitivity scale. All participants provided informed consent online and were debriefed and compensated at the conclusion of the study. The study procedures were approved by the University of Toronto Research Ethics Board (#31485).

### Procedure

Participants completed an online survey using Qualtrics software. Participants were asked questions about basic demographics, the extent to which they used and trusted various health services, institutions and professions, agreed with various statements relating to health attitudes and beliefs, attitudes towards vaccination, and levels of disgust sensitivity.

### Measures

#### Demographics and use of health services

A demographics form was provided asking participants their age, sex, level of education, political ideologies, level of religiosity, and use of health services. Level of education was determined with a 9-point scale of the following options: “No formal education”, “Some high school”, “High school diploma or equivalent”, “Some College”, “College Diploma/Degree”, “Some University”, “Bachelor’s Degree”, “Master’s Degree”, “PhD”. Political ideology was ranked on a 7-point Likert scale from “Strongly Liberal” to “Strongly Conservative” with higher scores indicating greater conservatism. Religiosity was assessed on a 7-point Likert scale with higher scores indicating greater religiosity. Finally, the degree to which participants use and rely on various health services, trust health services, treatments, and tests, and trust institutions and professions was assessed. These questions were administered on a 7-point Likert scale with responses ranging from least (0) to most (7). The full list of health services items can be found in the supporting information (S1 File).

### Attitude scales

#### Parent Attitudes about Childhood Vaccines Scale (PACV)

The *Parent Attitudes about Childhood Vaccines Scale (*PACV) [38] was used to define parental vaccine hesitancy in our cohort. Responses are scored from 0–100, with higher scores indicating greater vaccine hesitancy. A score of 50 or greater indicates a vaccine hesitant parent and is predictive of childhood immunization [39]. Examples of items include: “How concerned are you that any one of the childhood shots might not be safe?” and “It is my role as a parent to question vaccination.”

#### Three Domains of Disgust Scale (TDDS)

*Three Domains of Disgust Scale (*TDDS) [40] measures three factors of disgust sensitivity: pathogen, sexual, and moral disgust (with a subscale corresponding to each factor). Each subscale contains seven items, for a total of 21 items. Participants are asked to rate how disgusting they find the events described in each item on a 7-point scale from “not at all disgusting” to “extremely disgusting,” with higher values indicating more disgust. Examples of items include: “seeing a cockroach run across the floor” (pathogen subscale), “hearing two strangers having sex” (sexual subscale), and “cutting to the front of a line to purchase the last few tickets to a show” (moral subscale).

### Statistical analyses

All statistical analyses were performed in R 3.6.1 [41]. To test the hypothesis that mistrust in medicine and greater disgust sensitivity predicted higher parental vaccine hesitancy, we conducted a linear regression analysis. PACV scores were used as the measure of parental vaccine hesitancy. The global trust in medicine score was created by combining trust in physicians, surgeons, and hospital subscales, while the global disgust sensitivity score was created by combining the pathogen, sexual, and moral disgust subscales. Trust in medicine, disgust sensitivity, religiosity, and political affiliation were included as model predictors, while adjusting for the effects of age, sex, and level of education.

A secondary analysis was conducted to better understand the relationship between global disgust and medical trust between participants who scored on the highest and lowest ends of vaccine hesitancy. Participants who scored in the 25^th^ percentile or below, with scores ranging from 0–9, on the PACV were termed “Low PACV” (n = 119) while participants scoring in the 75^th^ percentile or above, with scores ranging from 50–94 were termed “High PACV” (n = 120). Prior to all analyses, Levene’s tests were conducted to check for homogeneity of variances. Based on these results, independent-subjects t-tests were used to analyze all disgust measures and Mann-Whitney U tests were used to analyze all medical trust measures.

## Results

After exclusions, the study included 484 individuals. The sample included 326 women (67.4%), 156 men (32.2%), and 2 participants (0.4%) who did not disclose their sex. Participants ranged in age from 19 to 77 years (*M = 42*.*09*, *SD = 11*.*40*). Additional participant characteristics can be found in Table 1.

**Table 1:** Participant characteristics.

	Women (n = 326)	Men (n = 156)	Total (n = 484)
**Sex (n (%))**			
**Women**	326 (67.4%)	--	--
**Men**	--	156 (32.2%)	--
**Declined to answer**	--	--	2 (0.4%)
**Education (n (%))**			
**No formal education**	3 (0.9%)	1 (0.6%)	4 (0.8%)
**Some high school**	30 (9.2%)	14 (9.0%)	44 (9.1%)
**High school diploma or equivalent**	89 (27.3%)	28 (17.9%)	118 (24.4%)
**Some college**	43 (13.2%)	17 (10.9%)	60 (12.4%)
**College diploma/degree**	11 (3.4%)	4 (2.6%)	15 (3.1%)
**Some university**	104 (31.9%)	65 (41.7%)	169 (34.9%)
**Bachelor’s degree**	41 (12.6%)	18 (11.5%)	59 (12.2%)
**Master’s degree**	1 (0.3%)	1 (0.6%)	2 (0.4%)
**PhD**	4 (1.2%)	8 (5.1%)	12 (2.5%)
**Declined to answer**	--	--	1 (0.2%)
**Age (M ± SD)**	41.66 ± 11.28	43.05 ± 11.65	42.09 ± 11.40
**Religiosity (M ± SD)**	3.97 ± 2.30	3.61 ± 2.37	3.85 ± 2.33
**Political affiliation (M ± SD)**	3.80 ± 1.75	4.20 ± 1.77	3.93 ± 1.76

M and SD are used to represent mean and standard deviation respectively.

**Fig 1:**
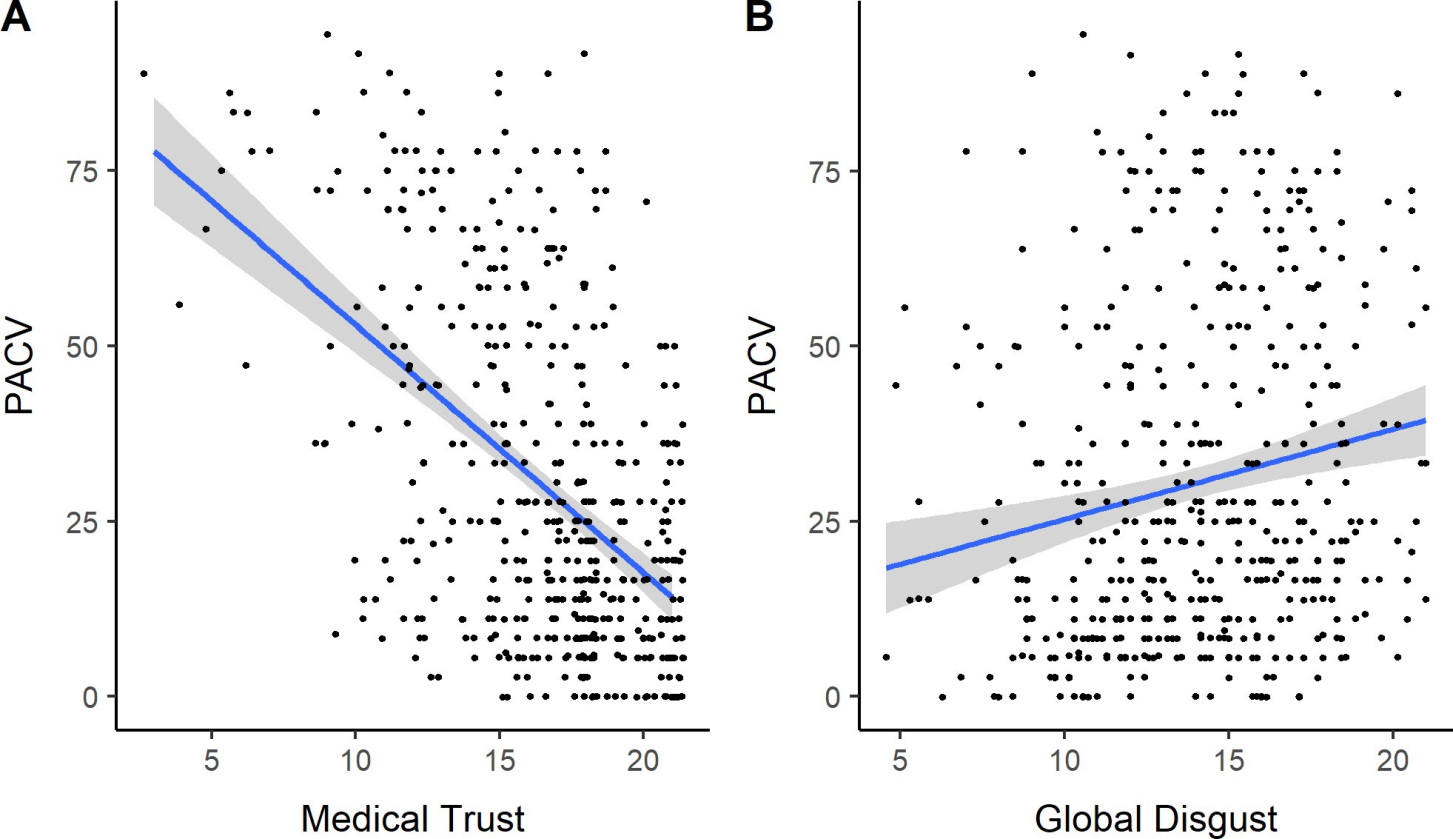
PACV scores over trust in medicine (A) and global disgust sensitivity (B).

We found significant relationships between greater vaccine hesitancy and higher levels of religiosity (*b = 1*.*32*, *SE =* .*51*, *p =* .*01*), younger age (*b = -*.*24*, *SE =* .*09*, *p =* .*006*), and lower levels of education (*b = -1*.*58*, *SE =* .*55*, *p =* .*004*) (Table 2). We also found that trust in medicine was the most significant predictor of PACV scores, with lowered trust in medicine predicting greater vaccine hesitancy *(b = -3*.*54*, *SE =* .*28*, *p <* .*001*) (Fig 1). A secondary regression revealed that decreased trust in physicians was the primary predictor of parental vaccine hesitancy *(b = -7*.*85*, *SE = 1*.*39*, *p <* .*001)*. Additionally. we found that global disgust sensitivity significantly predicted PACV scores, with greater disgust sensitivity being associated with higher vaccine hesitancy (*b =* .*95*, *SE =* .*35*, *p =* .*006)*. A secondary regression revealed that higher scores in the sexual disgust subscale was the primary predictor of parental vaccine hesitancy *(b = 1*.*87*, *SE =* .*86*, *p =* .*03)*.

**Table 2:** Linear regression of global disgust sensitivity and trust in medicine on parental vaccine hesitancy.

Predictor	*b*	*SE*	*t*	*p*	*r*
**Global disgust**	0.95*	.35	2.76	.006	.13
**Medical trust**	-3.54*	.28	-12.52	< .001	.51
**Religiosity**	1.32*	.51	2.58	.01	.12
**Political affiliation**	-0.11	.61	-0.18	.85	.01
**Age**	-0.24*	.09	-2.78	.006	.13
**Education**	-1.58*	.55	-2.90	.004	.14
**Sex**	-0.87	2.23	-0.39	.70	.02

*b* represents unstandardized regression coefficient. *SE* represents the standard error. *t* represents the t-statistic.

* indicates *p* < .05. *r* represents the Pearson correlation coefficient.

**Fig 2:**
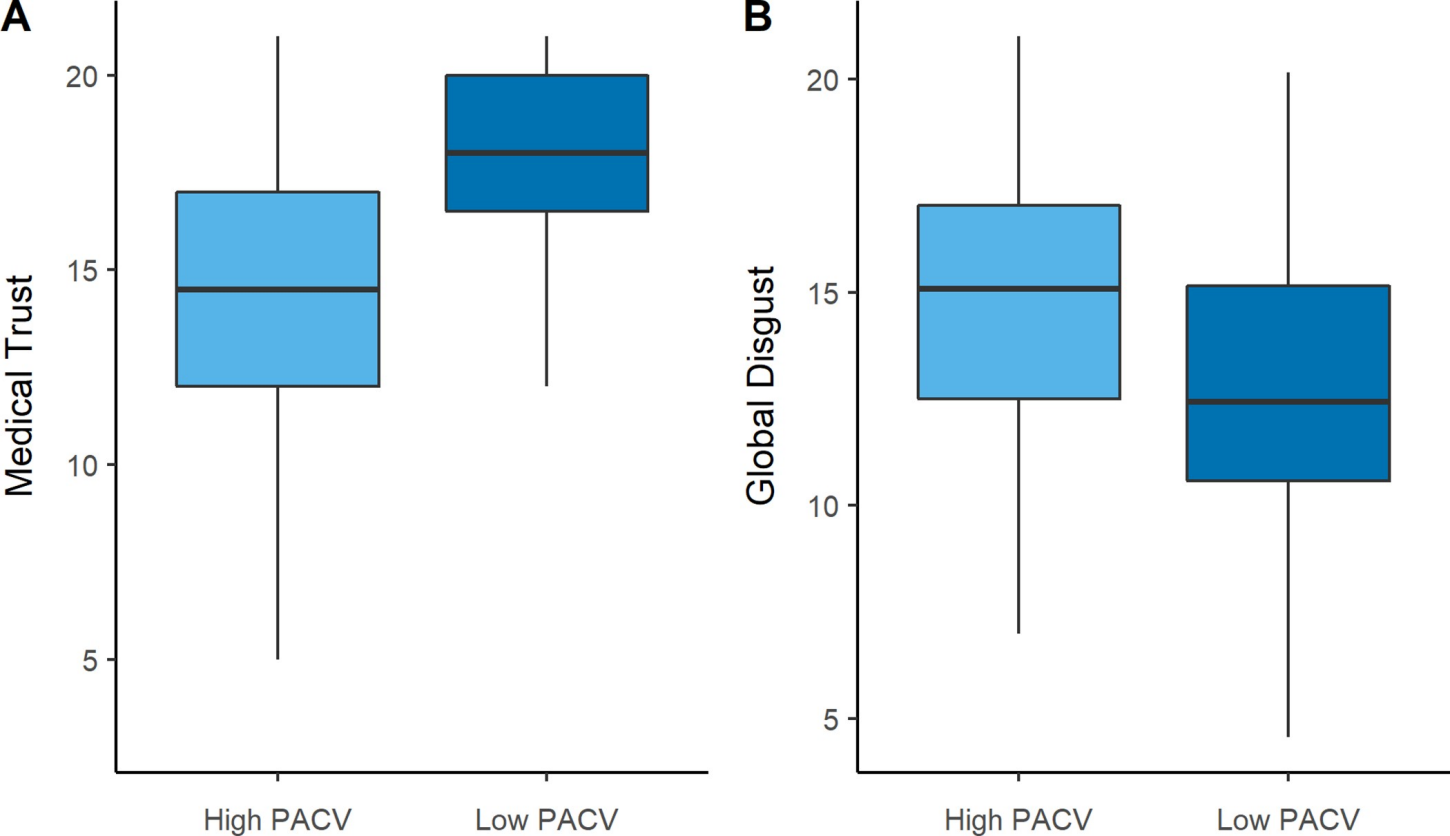
Trust in medicine (A) and global disgust sensitivity (B) between participants scoring high (<25th percentile) and low (>75th percentile) on the PACV.

Secondary analyses revealed that the High PACV group indicated significantly lower trust in medicine *(U = 2509*, *p <* .*001*), and greater global disgust *(t = 4*.*59*, *p <* .*001*) (Fig 2). Within the medical trust subscales, trust in physicians *(U = 2490*, *p <* .*001)*, surgeons *(U = 3425*.*5*, *p <* .*001)*, and hospitals *(U = 3266*, *p <* .*001)* were all significantly lower for the High PACV group. For the disgust subscales, pathogen disgust *(t = 2*.*74*, *p =* .*006)* and sexual disgust *(t = 5*.*80*, *p <* .*001)* were significantly higher in the High PACV group, with moral disgust showing a trend in this direction *(t = 1*.*56*, *p =* .*12)*.

## Discussion

We found that parental vaccine hesitancy was associated with younger age, greater religiosity, lower level of education, lower trust in physicians, and higher disgust sensitivity. Although these have been studied independently in previous work, ours is the first study to examine these factors within the same cohort, creating a better understanding of the strongest predictors of parental vaccine hesitancy.

Three demographic factors predicted higher vaccine hesitancy: age, religiosity, level of education. Similar to previous findings in a mixed-sex sample of Croatian parents, younger age predicted greater likelihood of vaccine hesitancy [42]. Younger parents are more likely to obtain parenting advice from the internet [43], where they may be exposed to online misinformation about vaccines [26]. Aligned with previous results from a national survey of Canadian parents [11], we also found that lower levels of education predicted greater vaccine hesitancy. Lower levels of education have been associated with reduced online heath literacy [44], which may provide an additional challenge in discerning what is true and untrue about online vaccine information. Finally, we found that higher religiosity predicted parental vaccine hesitancy. Religious beliefs are a legal avenue for personal vaccine exemptions, and some religious groups do not support the use of vaccines, so it is unsurprising that more religious parents are likely to be vaccine hesitant. We did not find a sex difference in parental vaccine hesitancy. However, women comprised two-thirds of our sample, which may reflect who makes the vaccine decisions in the family. To this point, several studies have focused exclusively on mothers’ vaccination decisions [22, 30, 45].

The results from this analysis demonstrate a strong relationship between mistrust of physicians and increased parental vaccine hesitancy. These findings are consistent with previous qualitative and descriptive research underlining the importance of trust in the parent-physician relationship for vaccine decision-making [19, 21–22, 45]. This suggests that pro-vaccine campaigns that rely on assumed trust in physicians may be ineffective for parents who are vaccine hesitant. For example, in 2019 the Ontario Medical Association launched a public education campaign designed to reduce vaccine hesitancy called #AskOntarioDoctors, with taglines such as “Vaccine questions? Ask your doctor–not the internet” (Ontario Medical Association, 2019). Despite being reasonable advice, a different approach may be needed for those whose vaccine hesitancy is linked to a lack of trust in physicians. Instead, some suggest medical providers and public health agencies aim to build trust with parents by using a combination of scientific fact and storytelling—adopting the narrative strategies often used on anti-vaccination sites [46]

Our results also found an association between greater disgust sensitivity and increased parental vaccine hesitancy. The relationship between disgust sensitivity on vaccine hesitancy in parents specifically has not been previously examined. Prior work has shown an association between pathogen disgust and vaccine hesitancy [32]. However, our results found the strongest association with sexual disgust rather than pathogen disgust. Sexual disgust in a U.S. sample has previously been shown to predict political conservatism, affiliating with the Republican Party, and voting for Donald Trump in the 2016 U.S. presidential election [47]. However, political conservatism was not found to be a predictor of parental vaccine hesitancy in this study, and it is unlikely that political conservatism is driving the association between sexual disgust and parental vaccine hesitancy.

It may be that sexual disgust is a unique predictor of parental vaccine hesitancy, and future research should continue to explore this concept. However, an alternative explanation may be that sexual disgust is reflecting a more robust measure of general disgust sensitivity. Of the three subscales, sexual and pathogen disgust had the highest test-retest reliability, and pathogen disgust was shown to have a specific association with contamination-based concerns [48]. Considering that vaccine hesitancy is multifaceted, and an individual may be vaccine hesitant for a variety of reasons, it may be that sexual disgust is capturing a more generalized disgust response beyond strictly contamination-based concerns.

Our finding of higher disgust sensitivity in vaccine hesitant parents shares features in common with work examining it in relation to Moral Foundations Theory, which proposes that moral decisions are often made using quick and emotional judgements, with post-hoc justifications to follow [49]. Previous work has shown vaccine hesitant parents strongly emphasize the moral foundations of purity and liberty [50]. Through this lens, vaccines violate purity, as they contain unnatural toxins. They also curtail liberty, as they are seen to violate civil liberties and impose government control. Importantly, disgust is seen to guard the body and soul, and forms the basis of the moral foundations of purity [49]. Thus, if disgust is the emotion associated with having one’s moral foundations violated, vaccine hesitant parents with higher disgust sensitivity may also perceive vaccines to be greater violations of purity and liberty.

However, this greater likelihood for higher disgust sensitivity may potentially be used to reduce vaccine hesitancy. In Horne et al. [14], vaccine hesitancy was reduced after exposure to photographs of children with measles and rubella—stimuli that could be considered disgust-inducing. By utilizing disgust-inducing stories and pictures to demonstrate the risks of not vaccinating, vaccine hesitant parents with higher disgust sensitivity may instead be more inclined to vaccinate their children.

## Limitations

The participants in this study were recruited through Amazon MTurk. Although research assessing the generalizability of MTurk has determined it to be a heterogeneous participant pool [51], our participants are not a representative sample of the population. A recent study determined MTurk workers to be more likely to be under age 50, more likely to have completed a college degree, and less likely to be vaccinated for influenza or to report being in excellent or very good health [52]. While these factors are limitations on the generalizability of our findings, our sample does reflect a representative distribution of age, education, general health, and the proportion of participants who did (n = 249) and did not (n = 243) receive a flu shot in the last season. Furthermore, this study required internet access to complete, which limits the generalizability of these results to the sub-population of internet users. However, anti-vaccination messaging is prevalent online [25], and vaccine hesitant parents are more likely to be internet users [23]. Additionally, this study was written in English, and included participants in the largely English-speaking countries of the United States, Canada, and the United Kingdom. Thus, the findings are limited to these countries. Importantly, vaccine hesitancy is a nuanced concept, and may be expressed in a variety of ways. One may hold vaccine-hesitant attitudes, yet still accept, delay, or refuse any combination of some or all vaccines. Although the PACV is a measure of parental vaccine hesitancy, it does not directly measure vaccine-related outcomes. Therefore, these results cannot capture possible demographic or attitudinal differences in the behavioral outcomes of parental vaccine hesitancy. Finally, the participants were all parents, as the outcome measure was specific to parental attitudes, and therefore may not necessarily reflect vaccine hesitancy in those who do not have children. However, understanding parents’ attitudes toward vaccination is of particular importance in devising strategies to ensure that children are vaccinated.

## Conclusions

Counteracting vaccine hesitancy and refusal will require a multifaceted effort, and a better understanding of key demographic and attitudinal predictors will be required for an effective approach. The results from this study suggest that the vaccine hesitant parent is of younger age, lower educational attainment, and greater religiosity with little trust in their physician and higher in disgust sensitivity. Taken together, these attributes begin to paint a picture of who is vaccine hesitant, and provide further insight into the demographic and attitudinal predictors needed to create successful public health messaging.

## Supporting information

S1 File Additional questions. (DOCX)
