# Supplementary material for: Mistrust of the medical profession and higher disgust sensitivity predict parental vaccine hesitancy
Source: PLoS One. 2020 Sep 2;15(9):e0237755. doi: 10.1371/journal.pone.0237755 (PMC7467323; doi:10.1371/journal.pone.0237755)
Supplement: S1 File — (DOCX) [file pone.0237755.s001.docx]

**S1 File. Additional questions.**

*Usage and reliance on health services*

Participants were asked to report the degree to which they use and rely on various health services. Health services included family doctor, pediatrician, massage therapy, chiropractor or osteopath, Chinese or Asian medicine, naturopath, homeopathic treatment, flu shots, vaccinations given to children to prevent childhood diseases, meditation, mindfulness, and medical marijuana.

*Trust in health services, treatments, and tests*

Participants were asked to indicate the degree to which they trust various health services, treatments, and tests. This was assessed for: natural medicines for cancer, vaccination for MMR, vaccination for HPV, chemotherapy for cancer, mammograms, colonoscopy, medication for mental illness, PSA test for prostate cancer, and psychotherapy.

*Trust in institutions and professions*

Participants reported the degree to which they trust the following institutions and professions: alternative medicine health providers, hospitals, scientists, psychiatrists, politicians, police, federal government, local government, physicians, dieticians, and surgeons.
